# AJCC 8th edition prognostic staging provides no better discriminatory ability in prognosis than anatomical staging in triple negative breast cancer

**DOI:** 10.1186/s12885-019-6494-3

**Published:** 2020-01-06

**Authors:** Jiehua He, Julia Y. Tsang, Xiaodan Xu, Jibin Li, Mei Li, Xue Chao, Yuanyuan Xu, Rongzhen Luo, Gary M. Tse, Peng Sun

**Affiliations:** 1 Department of Pathology, Sun Yat-sen University Cancer Center, Guangzhou, China; 2 State Key Laboratory of Oncology in South China, Guangzhou, China; 3 Collaborative Innovation Center for Cancer Medicine, Guangzhou, China; 4 Department of Anatomical and Cellular Pathology, Prince of Wales Hospital, The Chinese University of Hong Kong, Hong Kong, China; 5 Department of Physiology, Zhongshan School of Medicine, Sun Yat-sen University, Guangzhou, China; 6 Department of Clinical Research, Sun Yat-sen University Cancer Center, Guangzhou, China

**Keywords:** Triple negative breast cancer, AJCC 8th, Prognostic stage, Anatomic stage

## Abstract

**Background:**

We retrospectively compared the prognostic value between the AJCC 8th edition anatomic (AS) and prognostic staging (PS) system for triple negative breast cancer (TNBC) in a cohort from two involved institutions and a large population database.

**Methods:**

Clinicopathological data of TNBCs were identified in two involved institutions (SYSUCC-PWH cohort). Data from SEER database during 2010–2015 was also accessed. We restaged all cases into AS and PS group according to the AJCC 8th staging system.

**Results:**

A total of 611 and 31,941 TNBCs were identified in two cohorts, with a median follow-up of 53.5 and 27 months respectively. PS upstaged 46.1% of patients in SYSUCC-PWH cohort, and 62.4% in SEER cohort. No significant difference was observed in C index between AS and PS models for disease-specific survival (DSS), progression-free survival (PFS) or overall survival (OS) in either cohort. χ2 statistic and Hazard Ratio for PFS, DSS and OS showed better discrimination between IA and IB, IIB and IIIA, IIIA and IIIB in AS model than PS model. Besides, patients with IIIC unchanged stage showed worse PFS compared to those with AS IIIA or IIIB upstaged to PS IIIC in both cohorts(*p* = 0.049, *p* < 0.001).

**Conclusions:**

Our findings demonstrated that prognostic staging system did not provide better discriminatory ability in predicting TNBCs prognosis than anatomic staging system.

## Background

Cancer staging helps clinicians to determine prognosis and design treatment plans for individual patient. Since 1977, the American Joint Committee on Cancer (AJCC) staging system for breast cancer (BC) has assigned stage based on anatomical parameters, including tumor size and extent of tumors (T), Lymph node involvement (N) and the presence or absence of distant metastasis (M). Once the T, N, and M are determined, they are combined for assigning the overall anatomic stage (AS) of 0, I, II, III, IV. However, a significant limitation of AS for BCs is that it does not include the biologic factors and may not represent the BC biologic behavior sufficiently.

Identification of BCs molecular subtypes and significant progress in genomics studies have led to a better understanding of BCs biologic behavior, which provides valuable information for the individualized treatment of BCs. Therefore, AJCC 8th Edition Cancer Staging Manual has updated with a prognostic stage (PS) group for breast cancer by incorporating traditional TNM anatomic parameters and additional biologic factors, including estrogen receptor (ER) and progesterone receptor (PR) status, HER2 status, tumor grade and Recurrence Scores (Oncotype Dx), which are known to have predictive and prognostic value [1, 2]. Previous validation studies have demonstrated that AJCC 8th edition PS provided more accurate prognostic information than AS in HR positive and HER2 positive breast cancer [3–6]. However, for triple negative breast cancer (TNBC) patients that lack expression of ER, PR and HER2, whether PS exhibits a better prognostic value than AS in these patients is still debatable [7, 8].

In this study, we retrospectively compared the prognostic value between the AJCC 8th edition AS and PS for TNBCs in a large local cohort (Sun Yat-sen University Cancer Center and Prince of Wales Hospital) and performed a SEER population-based analysis. We aim to examine whether this novel PS system provide more accurate risk stratification in overall survival, disease-specific survival, progression-free survival than the AS system.

## Methods

### Identification of SYSUCC-PWH cohort

Clinicopathologic data, including tumor histologic grade, pathologic T and N categories, and ER, PR, and HER2 status, for patients who underwent surgery as the initial intervention with pathological confirmed invasive breast cancer in Sun Yat-sen University Cancer Center (SYSUCC) during 2005–2013 and Prince of Wales Hospital (PWH) during 2002–2008 were recorded. Patients receiving neoadjuvant therapy, and those with stage IV disease, re-operation for local recurrence,or incomplete or unknown clinicopathologic data were excluded.

All pathologic specimens were reviewed by experienced pathologists to confirm the clinicopathologic characteristics including the tumor size, axillary nodal status, resection margin. ER, PR, and HER2 status were determined according to immunohistochemical (IHC) staining. For patients before 2010, ER and PR status was reclassified as negative using a cut-off of 1% according to the American Society of Clinical Oncology/College of American Pathologists (ASCO/CAP) guidelines [9]. HER2 status was define as negative with 0, 1+ on IHC as well as 2+ on IHC without HER2 gene amplification on fluorescence in situ hybridization (FISH) [10].

### Identification of SEER cohort

The SEER 18 Registry Research Data was accessed through the SEER*stat (Version 8.3.5) to extract cases in this cohort [11]. Since the HER2 status was not available for cases before 2010, the cases from SEER database was limited to the duration during 2010–2015. “Breast” was selected in side recode ICD-O-3/WHO 2008 category and “Triple Negative” was selected in Breast Subtype (2010+) category. Clinicopathologic data including age at diagnosis, sex, year of diagnosis, primary site, histologic type, histologic grade, AJCC 7th edition stage group (T, N, M), survival months, cause-specific death classification, treatment information, such as surgery, chemotherapy and radiotherapy, were collected. Cases with stage IV disease and those with incomplete or unknown clinicopathologic data were excluded.

### Statistical analysis

PS and AS were determined for cases in both SYSUCC-PWH cohort and SEER cohort according to the AJCC 8th edition staging manual. Cases with histologic grade of grade III (poorly differentiated) and IV (Undifferentiated; anaplastic) in SEER cohort were both classified as G3 in PS system. Distributions of AS and PS were compared in each cohort. We evaluated the number of patients with alterative stage by the PS system. PFS was define as the duration between date of diagnosis to date of disease progression or last contact. DSS was define as the duration between date of diagnosis to date of death because of breast cancer progression or last contact. OS was define as the duration between date of diagnosis to date of death or last contact. PFS and DSS according to AS and PS were compared for SYSUCC-PWH cohort. OS and DSS between the stages were compared for SEER cohort.

The log-rank test was used to compare differences of survival rates between groups. The Harrell concordance index (C index) was calculated using SAS (Version 9.4) to measure the model’s predictive performance for the AS and PS models. A higher C index indicates a better predictive performance. Significance between the C index of AS and PS models was determined using *t* test. The χ2 statistic of the log-rank test was used to further calculate the discrimination between groups. A larger χ2 statistic indicates further distance between survival curves, and its *P* value reflects the statistical significance of this distance. The relationship of AS and PS with DSS, PFS or OS was modeled using a Cox proportional hazards regression model. The results were expressed in hazard ratios (95% CIs). Two-tailed *p* value < 0.05 was considered statistically significant, and IBM SPSS (Version 21.0) was used for analysis.

## Results

A total of 611 patients with stage I to IIIC TNBC were identified in the SYSUCC-PWH cohort, of which 427 and 184 patients were from Sun Yat-sen University Cancer Center (SYSUCC) and Prince of Wales Hospital (PWH) respectively. All patients were assigned a PS. Compared to AS, PS upstaged 282 patients (46.1%) and, unchanged stage was in 329 patients (53.9%). No patient showed downstaged PS in this cohort. For those with upstaged PS, 232 patients (232/282, 82.3%) showed one stage up (including IA to IB, IIB to IIIA, IIIA to IIIB and IIIB to IIIC) and 50 patients (50/282, 17.7%) showed two stages up (IIIA to IIIC).

A total of 31,941 patients with stage I to IIIC TNBC were identified in the SEER cohort. Compared to AS, PS upstaged 19,924 patients (62.4%), and unchanged stage was in 12,009 patients (37.6%). Eight patient showed downstaged PS (IIIC to IIIB) in this cohort. For those with upstaged PS, 17771 out of 19,924 patients (89.2%) showed one stage up (including IA to IB, IIB to IIIA, IIIA to IIIB and IIIB to IIIC) and 2153 patients (10.8%) showed two stages up (IIIA to IIIC). Clinicopathologic characteristics and staging alterations for patients in both cohorts are listed in Additional file 3: Tables S1 and S2.

**Fig. 1:**
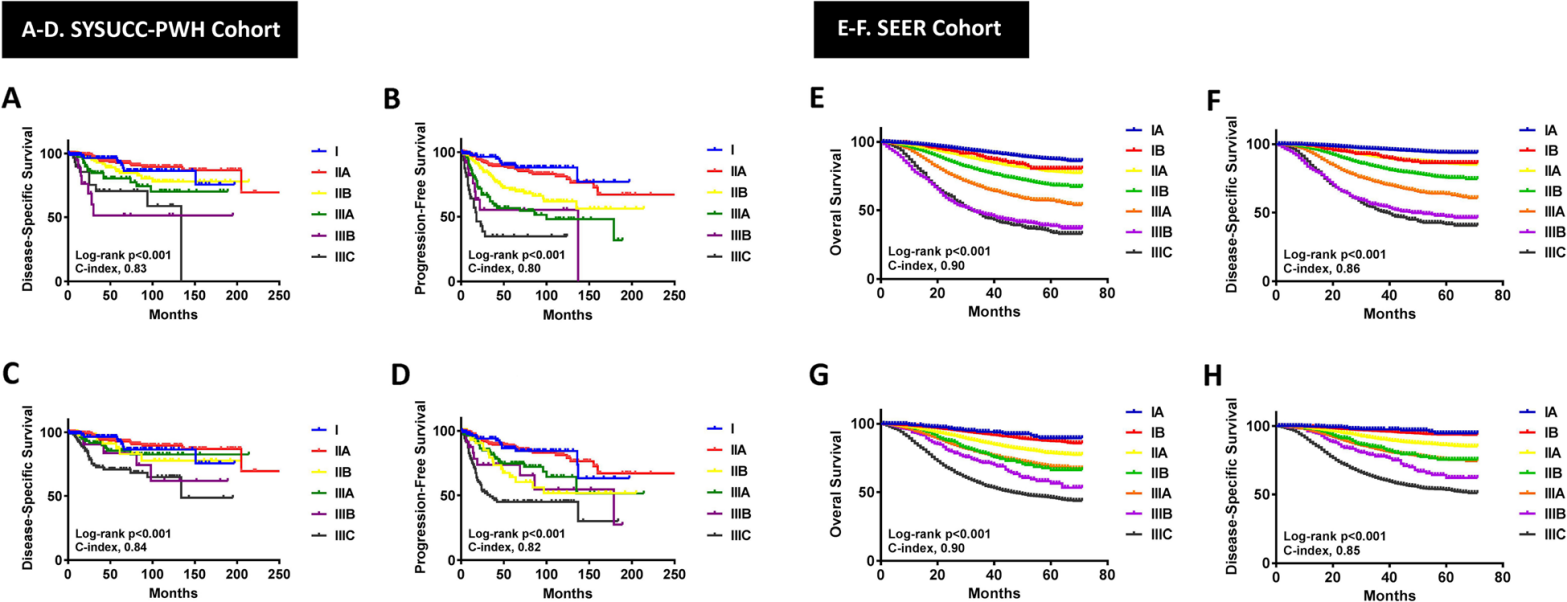
Kaplan-Meier curves of DSS and PFS for the SYSUCC-PWH cohort in anatomical staging system (**a**-**b**) and prognostic staging system (**c**-**d**). Kaplan-Meier curves of OS and DSS for the SEER cohort in anatomical staging system (**e**-**f**) and prognostic staging system (**g**-**h**)

Survival analyses were conducted in both cohorts and survival curves are shown in Fig. 1. The median follow-up was 53.5 months for the SYSUCC-PWH cohort and 27 months for the SEER cohort. Five-year DSS and PFS for SYSUCC-PWH cohort by AS and PS are summarized in Table 1. Five-year DSS and OS for SEER cohort by AS and PS are summarized in Table 2. In SYSUCC-PWH cohort, the C index for AS was 0.83 (95% CI 0.63–0.98) in DSS and 0.80 (95% CI 0.59–0.96) in PFS. The C index for PS was 0.84 (95% CI 0.63–0.98) in DSS and 0.82 (95% CI 0.62–0.97) in PFS (Fig. 1). No significant difference of C index between AS and PS models, reflecting that PS model may not be a more predictive accurate model for either DSS (*p* = 0.943) or PFS (*p* = 0.887) than AS model. Similar outcomes were observed in SEER cohort, the C index for AS was 0.86 (95% CI 0.81–0.89) in DSS and 0.90 (95% CI 0.87–0.92) in OS. The C index for PS was 0.85 (95% CI 0.81–0.89) in DSS and 0.90 (95% CI 0.87–0.92) in OS. No significant difference of C index between AS and PS models again in either DSS (*p* = 0.95) or OS (*p* = 0.98).

**Table 1:** 5-year Progression-Free Survival and Disease-Specific Survival by Anatomic and Prognosis Stage of TNBCs included in SYSUCC-PWH cohort (*N* = 611)

	Stage	No. at risk	5-year PFS (95% CI)	*p*-value	No. at risk	5-year DSS (95% CI)	*p*-value
AS	I(A*)	100	86.5(76.2,92.5)	< 0.001**	100	92.9(83.4,97.0)	0.001**
	II	371	82.0(77.0,86.0)	0.001^#^	371	90.6(86.3,93.6)	0.080^#^
	IIA	240	87.9(82.4,91.9)		240	92.7(87.7,95.7)	
	IIB	131	71.0(60.9,79.0)		131	86.2(76.8,92.0)	
	III	140	50.9(41.1,59.8)	0.045^△^	140	74.1(64.2,81.7)	0.099^△^
	IIIA	78	56.6(43.7,67.6)		78	80.4(67.8,88.5)	
	IIIB	29	55.2(28.6,75.4)		29	51.4(23.1,73.9)	
	IIIC	33	34.9(18.3,52.2)		33	70.4(46.9,85.0)	
PS	I	100	86.5(76.2,92.5)	< 0.001**	100	92.9(83.4,97.0)	< 0.001**
	IA	3	NA	NA	3	NA	NA
	IB	97	86.0(75.4,92.3)		97	92.7(88.5,97.0)	
	II	291	83.9(78.3,88.2)	0.002^#^	291	91.9(87.2,94.9)	0.209^#^
	IIA	240	87.9(82.4,91.9)		240	92.7(89.7,95.7)	
	IIB	51	64.1(46.1,77.5)		51	87.3(79.4,95.1)	
	III	220	60.1(52.3,66.9)	< 0.001^△^	220	78.2(70.7,83.9)	0.023^△^
	IIIA	83	74.7(62.2,83.6)		83	85.5(78.7,92.3)	
	IIIB	26	73.7(50.4,87.3)		26	83.5(72.5,94.5)	
	IIIC	111	44.9(34.0,55.1)		111	70.7(61.8,79.7)	

**Log-rank test comparing proportions among all stage; ^#^Log-rank test comparing proportions among Stage II; ^△^Log-rank test comparing proportions among Stage III; *No case was classified as anatomic stage IB in this cohort

*TNBC* triple negative breast cancer, *AS* anatomic stage, *PS* prognosis stage, *PFS* progression-free survival, *DSS* disease specific survival, *CI* confidence interval

**Table 2:** 5-year Overall Survival and Disease-Specific Survival by Anatomic and Prognosis Stage of TNBCs included in SEER cohort (*N* = 31,941)

	Stage	No. at risk	5-year OS (95% CI)	*p*-value	No. at risk	5-year DSS (95% CI)	*p*-value
AS	I	12,700	87.2(86.2,88.0)	< 0.001**	12,700	93.5(82.8,94.2)	< 0.001**
	IA	12,293	87.4(86.5,88.3)	0.004*	12,293	93.8(93.1,94.4)	0.001*
	IB	407	80.4(73.9,85.5)		407	86.2(80.6,90.2)	
	II	14,292	75.3(74.2,76.4)	< 0.001^#^	14,292	82.7(81.8,83.7)	< 0.001^#^
	IIA	9713	78.5(77.2,79.7)		9713	86.0(84.9,87.0)	
	IIB	4579	68.3(66.1,70.4)		4579	75.5(73.5,77.4)	
	III	4949	46.8(44.8,48.7)	< 0.001^△^	4949	54.0(52.0,56.0)	< 0.001^△^
	IIIA	2515	57.1(54.3,59.7)		2515	63.6(60.8,66.2)	
	IIIB	1164	38.5(34.5,42.5)		1164	47.0(42.6,51.2)	
	IIIC	1270	34.3(30.5,38.2)		1270	41.4(37.3,45.2)	
PS	I	12,293	87.4(86.5,88.3)	< 0.001**	12,293	93.8(93.1,94.4)	< 0.001**
	IA	407	89.4(83.6,93.2)	0.212*	407	94.6(89.2,97.3)	0.259*
	IB	11,886	87.4(86.5,88.3)		11,886	93.8(93.1,94.4)	
	II	10,726	77.9(76.7,79.1)	< 0.001^#^	10,726	85.5(84.4,86.5)	< 0.001^#^
	IIA	10,120	78.6(77.3,79.8)		10,120	86.0(85.0,87.0)	
	IIB	606	67.4(61.2,72.9)		606	75.4(69.3,80.4)	
	III	8922	56.3(54.8,57.8)	< 0.001^△^	8922	63.5(62.0,65.0)	< 0.001^△^
	IIIA	3986	68.7(66.4,70.8)		3986	75.6(73.4,77.6)	
	IIIB	365	56.3(47.8,64.0)		365	62.1(53.0,70.0)	
	IIIC	4571	45.9(43.9,48.0)		4571	53.3(51.2,55.4)	

**Log-rank test comparing proportions among all stage;*Log-rank test comparing proportions among Stage I; ^#^Log-rank test comparing proportions among Stage II; ^△^Log-rank test comparing proportions among Stage III

*TNBC* triple negative breast cancer, *AS* anatomic stage, *PS* prognosis stage, *OS* overall survival, *DSS* disease specific survival, *CI* confidence interval

The χ^2^ statistic was applied to evaluate the discriminatory power between different stages among AS and PS in both cohorts. In the SYSUCC-PWH cohort, χ^2^ on PFS between IIA and IIB for AS was 10.36 (*p* = 0.0013) and that for PS was similar, showing a χ^2^ of 10.05 (*p* = 0.0015). Comparing between IIB and IIIA, χ^2^ on PFS was 5.765 (*P* = 0.0163) for AS while that for PS was 0.6587 (*P* = 0.417). Similarly, between IIIA and IIIB, AS model also displayed a larger χ^2^ statistic than PS on DSS (χ^2=^4.204, *P* = 0.0403 vs χ^2=^1.239, *P* = 0.2656). χ^2^ statistic and *P* value on pair-wise stage comparison of the cohort are shown in Table 3.

**Table 3:** The χ2 statistic on Disease-Specific Survival and Progression-Free Survival for Anatomic and Prognostic Stage of TNBCs included in SYSUCC-PWH cohort (*N* = 611)

Stage	Disease-Specific Survival				Progression-Free Survival			
	AS		PS		AS		PS	
	χ2	*p*-value	χ2	*p*-value	χ2	*p*-value	χ2	*p*-value
I^a^ vs IIA	0.5876	0.4433	0.5876	0.4433	0.006753	0.9345	0.0068	0.9345
IIA vs IIB	3.214	0.073	1.572	0.2099	10.36	0.0013	10.05	0.0015
IIB vs IIIA	2.16	0.1417	0.0016	0.9681	5.765	0.0163	0.6587	0.417
IIIA vs IIIB	4.204	0.0403	1.239	0.2656	0.5332	0.4653	0.7811	0.3768
IIIB vs IIIC	0.2007	0.6541	0.7913	0.3737	1.922	0.1656	2.294	0.1299

^a^No case was classified as anatomic stage IB in this cohort

*TNBC* triple negative breast cancer, *AS* anatomic stage, *PS* prognosis stage

In the SEER cohort, compared to PS model, χ^2^ statistic on DSS of AS model showed a larger and statistically significant difference between IA and IB (24.94, *P* < 0.0001 vs 1.272, *P* = 0.2593), IIA and IIB (137.6, *P* < 0.0001 vs 23.93, *P* < 0.0001), IIB and IIIA (98.42, *P* < 0.0001 vs 0.249, *P* = 0.6178) as well as IIIA and IIIB (97.38 *P* < 0.0001 vs 9.91, *P* = 0.0016). However, PS model displayed larger χ^2^ statistic on DSS than AS model between IB and IIA (0.0045, *P* = 0.9461 vs 220, *P* < 0.0001) as well as IIIB and IIIC (0.68, *P* = 0.4088 vs 21.1, *P* < 0.0001). Similar results were also observed on the discrimination between stages for OS. χ^2^ statistic and *P* value on pair-wise stage comparison of the cohort are shown in Table 4.

**Table 4:** The χ2 statistic on Disease-Specific Survival and Overall Survival for Anatomic and Prognostic Stage of TNBCs included in SEER cohort (*N* = 31,941)

Stage	Disease-Specific Survival				Overall Survival			
	AS		PS		AS		PS	
	χ2	*p*-value	χ2	*p*-value	χ2	*p*-value	χ2	*p*-value
IA vs IB	24.94	< 0.0001	1.272	0.2593	8.254	0.0041	1.561	0.2115
IB vs IIA	0.0045	0.9461	220	< 0.0001	1.247	0.2641	183.3	< 0.0001
IIA vsIIB	137.6	< 0.0001	23.93	< 0.0001	109.5	< 0.0001	22.64	< 0.0001
IIB vs IIIA	98.42	< 0.0001	0.249	0.6178	92.07	< 0.0001	0.005236	0.9423
IIIA vs IIIB	97.38	< 0.0001	9.91	0.0016	128.1	< 0.0001	11	0.0009
IIIB vs IIIC	0.6823	0.4088	21.1	< 0.0001	0.0601	0.8063	21.44	< 0.0001

*TNBC* triple negative breast cancer, *AS* anatomic stage, *PS* prognosis stage

**Fig. 2:**
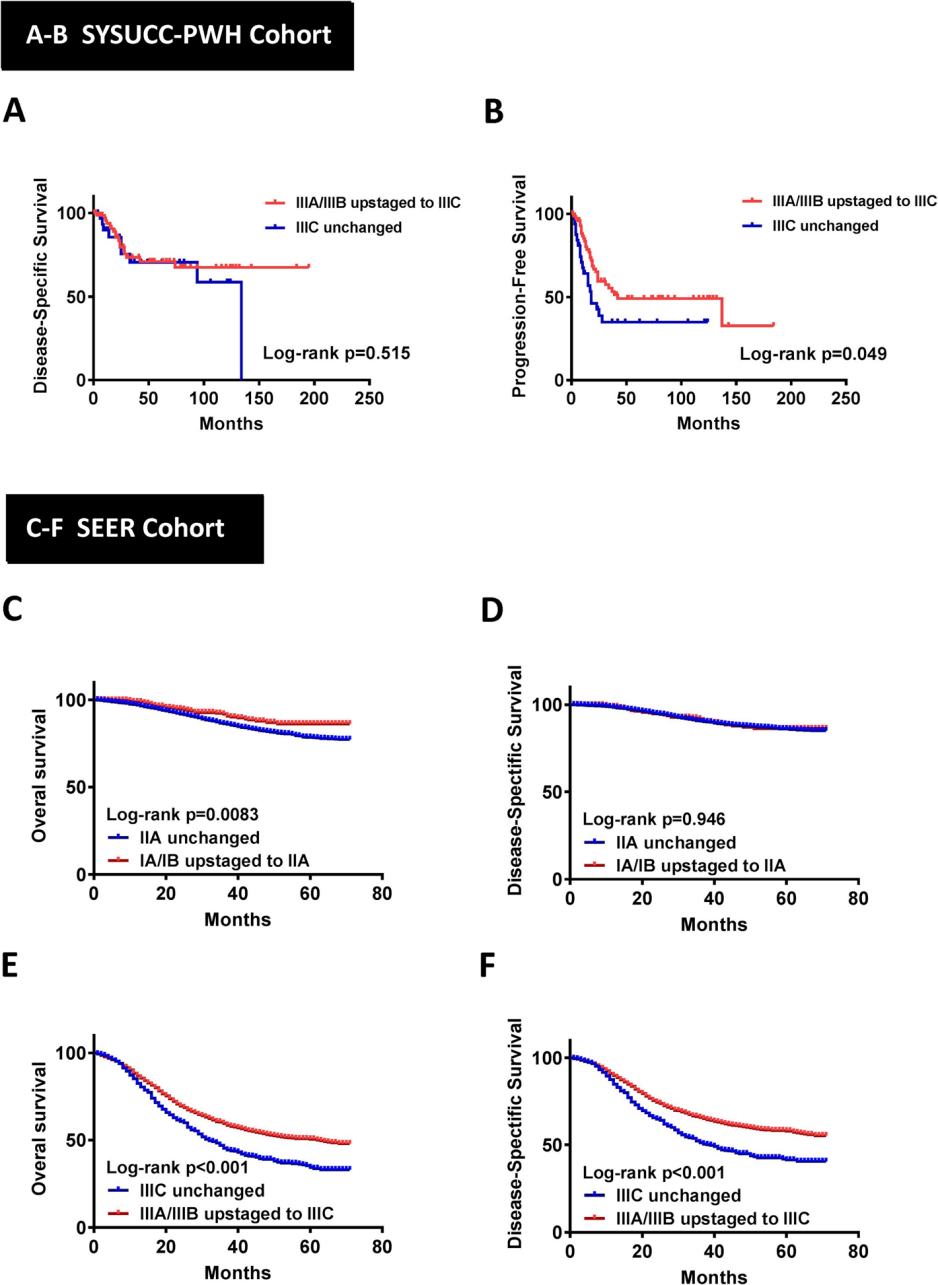
Kaplan-Meier curves of DSS (**a**) and PFS (**b**) for the SYSUCC-PWH cohort between patients with IIIC unchanged and those with anatomical stage IIIA/IIIB upstaged to prognostic stage IIIC. Kaplan-Meier curves of OS (**c**) and DSS (**d**) for the SEER cohort between patients with IIA unchanged and those with anatomical stage IA/IB upstaged to prognostic stage IIA; Kaplan-Meier curves of OS (**e**) and DSS (**f**) for the SEER cohort between patients with IIIC unchanged and those with anatomical stage IIIA/IIIB upstaged to prognostic stage IIIC

Furthermore, we also compare survival data between patients with unchanged stage and patients with upstaged PS. In SYSUCC-PWH cohort, patients with IIIC unchanged stage showed worse survival outcome than those patients with AS IIIA or IIIB upstaged to PS IIIC in PFS (*p* = 0.049, Fig. 2b), but not in DSS (*p* = 0.515, Fig. 2a). Similarly, in SEER cohort, patients with IIIC unchanged stage showed worse survival outcome in both OS and DSS when compared to patients with AS IIIA or IIIB upstaged to PS IIIC (*p* < 0.001, Fig. 2e-f). Besides, patients with IIA unchanged stage also showed worse OS than patients with AS IA or IB upstaged to PS IIA (*p* = 0.0083, Fig. 2c). On the other hand, we found no significant difference in either DSS, PFS or OS between patients with IIB unchanged stage and those with AS IIB upstaged to PS IIIA in both cohorts. Survival curve and *p* value are shown in Additional file 1: Figure S1 and Additional file 2: Figure S2.

A Cox proportional hazards regression model was then used to look into the Hazard Ratio (HR) for DSS, PFS or OS by stage in both cohorts. The hazard ratios for anatomic and prognostic stages in both cohorts are summarized in Additional file 3: Tables S2, S3 and S4.

## Discussion

The greatest change of AJCC 8th edition cancer staging system is the incorporation of tumor grade, and ER, PR, and HER2 status, as well as genomic assays, which are generally accepted as important predictive and prognostic factors in clinical practice [9–14]. This mean that prognostic stage is more likely to reflect individual prognosis when patients receive the recommended treatment strategies [1, 2, 6].

Previous studies have demonstrated that the PS model provided a better prognosis value than the AS model in individuals with breast cancer [3, 4, 6, 15], including the subgroup analysis for ER positive [5] and HER2 positive BCs [16]. This may due to the selection of genomic low risk patients and the improved effectiveness of endocrine therapy and anti-HER2 therapy. When looking into patients with TNBC, who predominantly receive chemotherapy, are more likely to have mid-high nuclear grade and worse prognosis for overall survival and disease-free survival [17, 18]. It is not surprising that, 46.1 and 62.4% TNBC patients were upstaged from AS to PS model in our two study cohorts. The upstaged rate in TNBC is greater than that reported for all breast cancer (6.8–34.7% approximately) [3, 4, 6, 15]. On the other hand, only 8 out of 31,941 patients with TNBC in SEER cohort were downstaged from AS IIIC to PS IIIB because of the low nuclear grade (grade 1). Compared to the reported downstaged rate of 23.4–35.6% approximately in overall breast cancer [3, 4, 6, 15], rarely patients with TNBC were downstaged in PS model.

Weiss, et al. [6] found that 13.6% BC patients could not be assigned to a prognostic stage due to the presence of N1mi disease in patientsnwith tumors larger than T1 or uncategorized combinations of T and N categories with grade and HR and HER2 status. Some subsequent changes had been made and demonstraded in the AJCC 8th Edition Updates and Corrections, that N1mi disease in patients with T2, T3 and T4 cancers includes N1mi. In the present study, all TNBCs were perfectly assigned to a proper prognostic stage according to the criterion. However, the results of present study in both TNBC cohorts do not show better prognostic value for PS model compared to the traditional AS model. PS model showed better discrimination for both OS and DSS between IB and IIA as well as IIIB and IIIC. However, worse discrimination between IA and IB as well as IIB and IIIA were found in PS compared to AS model. Those upstaged from AS (IIIA/IIIB to IIIIC) showed a significant worse survival than those with unchanged PS (IIIC). Similarly, those upstaged to a higher PS (e.g. IIB to IIIA) did not show significant worse survival than PS stage equivalent to their original AS (IIB unchanged). Our findings demonstrated that the new prognostic staging system do not provide better discriminatory ability in predicting TNBCs prognosis than anatomic staging system.

Upstaged TNBCs by PS in the present study are mostly from AS IA, IIB, IIIA and IIIB, however, we noticed that those upstaged cases do not demonstrate the relevant worse survival outcomes. These findings are consistent with previous study by Liu, et al. [8]. On the contrary, contradictory studies by Li, et al. [7] and Luo, et al. [19] indicated that the PS system displayed a more optimistic prognostic stratification and predictability than traditional AS system. However, Li, et al. [7] applied a earlier version of AJCC 8th criterion without subsequent corrections in a small sample cohort including stage IV disease. They also excluded special types of invasive breast cancer, and no relevant statistical methods had been applied to further assess and compare prognostic ability of the two staging systems. Luo, et al. [19] used the goodness-of-fit test, included statistics as −2likelihood, AIC, and BIC, to describe the prediction capability of the two competing staging systems in TNBCs and found that new version of AJCC staging system were higher than before. However, they also mentioned that statistics such as AIC and BIC are not convertible to a clinical meaningful relevance. The calculation of the C-index at different time points did not show significant differences in the two competing stage systems, which is in line with our observations.

The mainstay treatment of TNBC remains to be chemotherapy. Despite the upstages in PS, the clinical treatment decision will unlikely be changed for the time being [6, 20]. So it is unnecessary to worry about the overtreatments for TNBCs. However, PS could not accurately stratify risk in TNBC, thus further updates should be required. Additional morphological and genomic information with prognostic significance in TNBCs have been demonstrated. Upcoming studies should consider the incorporation of biologic factors that closely related to the development of novel clinical therapies in TNBCs, for example, Poly-ADP-ribose polymerase inhibitors (PARPi) including Olaparib or Talazoparib and platinum therapy may provided a significant benefit over standard chemotherapy with respect to progression-free survival for metastatic TNBCs with germline BRCA1/2 mutation [21–23]; Presence and increasing percentage of stromal tumor infiltrating lymphocytes (sTILs) [24, 25] are associated with better response to anthracycline-based neoadjuvant chemotherapy and improved long-term survival in TNBCs; Atezolizumab plus nab-paclitaxel prolonged progression-free survival among patients with metastatic triple-negative breast cancer in PD-L1-positive subgroup [26]. Moreover, activation PI3K/AKT signaling [27], AR expression [28], histopathological and molecular subtypes [29, 30] should also be considered for the modification of the prognostic staging system in TNBCs. One limitation of the present study is that we cannot acquire data regarding administration of PARPi and immunotherapy. As a retrospective study, we enrolled TNBCs diagnosed at the time during 2005–2015 (SYSUCC, 2005–2013; PWH, 2002–2008; SEER, 2010–2015), when PARPi and immunotherapy were not widely approved for clinical practice and the predominant treatment of TNBC remains to be chemotherapy and radiotherapy, especially in Asian countries. However, subsequent clinical trials, including EMBRACA [21] Trial, OlympiAD [22] Trial, PrECOG 0105 Trial [22] and IMpassion130 Trial [26], suggested the significant effects of novel treatment modalities especially for advanced TNBCs. Another limitation of the present study is that no case was classified as anatomic stage IB in SYSUCC-PWH cohort. In SEER cohort, we found that few cases of TNBC (1.3%) were classified as AS IB (T0NmiM0 and T1N1miM0). So it can be happened that no AS IB case was found in the SYSUCC-PWH cohort with a smaller sample size. This may also due to the reluctance for pathologists to make the diagnosis of Nmi at the time of initial diagnosis. However, all the AS IB cases, regardless of the variety in histologic grade, are classified as the unchanged PS IB following the AJCC 8th criteria, which may not affect outcomes of the comparative study.

## Conclusions

Our findings demonstrated that prognostic staging system did not provide better discriminatory ability in predicting TNBCs prognosis than anatomic staging system. The prognosis staging system should be further modified following the development of novel clinical therapies in TNBCs.

## Supplementary information

**Additional file 1: Figure S1.** Kaplan-Meier curves of DSS (A) and PFS (B) for the SYSUCC-PWH cohort between patients with IIB unchanged and those with anatomical stage IIB upstaged to prognostic stage IIIA.

**Additional file 2: Figure S2.** Kaplan-Meier curves of OS (A) and DSS (B) for the SEER cohort between patients with IA unchanged and those with anatomical stage IA upstaged to prognostic stage IB; Kaplan-Meier curves of OS (C) and DSS (D) for the SEER cohort between patients with IIB unchanged and those with anatomical stage IIB upstaged to prognostic stage IIIA.

**Additional file 3: Table S1.** Clinicopathologic characteristics of TNBCs included in SYSUCC-PWH cohort (*N* = 611) and SEER cohort (*N* = 31,941). **Table S2.** Alteration from Anatomic to Prognostic Stage of TNBCs included in SYSUCC-PWH cohort (*N* = 611) and SEER cohort (*N* = 31,941). **Table S3.** Hazard Ratio for Disease-Specific Survival and Progression-Free Survival by Stage of TNBCs included in the SYSUCC-PWH cohort (*N* = 611). **Table S4.** Hazard Ratio for Disease-Specific Survival and Overall Survival by Stage of TNBCs included in the SEER cohort (*N* = 31,941).

## Data Availability

The SEER-database is publicly available. The datasets generated and/or analysed during the current study are also available from the corresponding author on reasonable request.

## Abbreviations

AJCC: The American Joint Committee on Cancer
AS: Anatomic stage
BC: Breast cancer
DSS: Disease-specific survival
OS: Overall survival
PFS: Progression-free survival
PS: Prognostic stage
PWH: Prince of Wales Hospital
SEER database: Surveillance, epidemiology, and end results database
SYSUCC: Sun Yat-sen University Cancer Center
TNBC: Triple negative breast cancer
