# Supplementary figures and images for: AJCC 8th edition prognostic staging provides no better discriminatory ability in prognosis than anatomical staging in triple negative breast cancer
Source: BMC Cancer. 2020 Jan 6;20:18. doi: 10.1186/s12885-019-6494-3 (PMC6945658; doi:10.1186/s12885-019-6494-3)

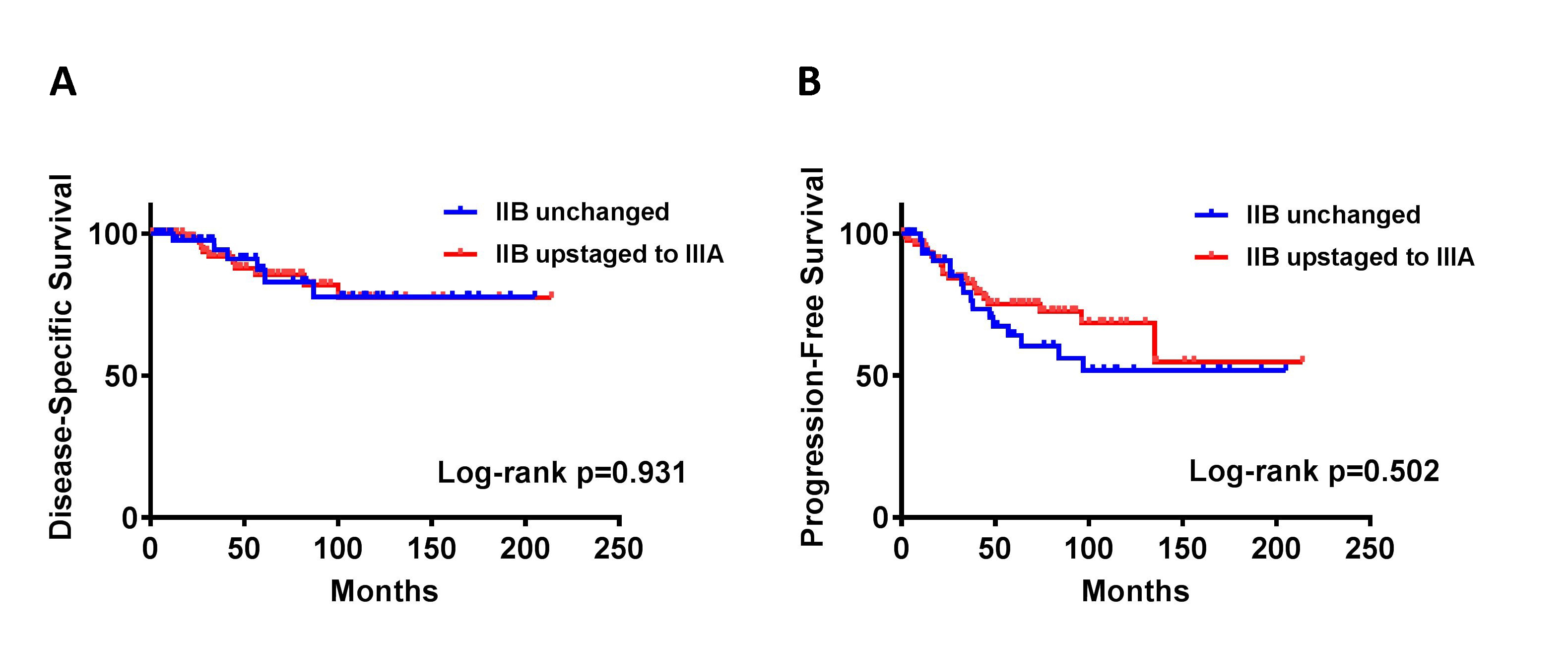

Supplement: Supplementary file 1 — Additional file 1: Figure S1. Kaplan-Meier curves of DSS (A) and PFS (B) for the SYSUCC-PWH cohort between patients with IIB unchanged and those with anatomical stage IIB upstaged to prognostic stage IIIA. [file 12885_2019_6494_MOESM1_ESM.jpg]

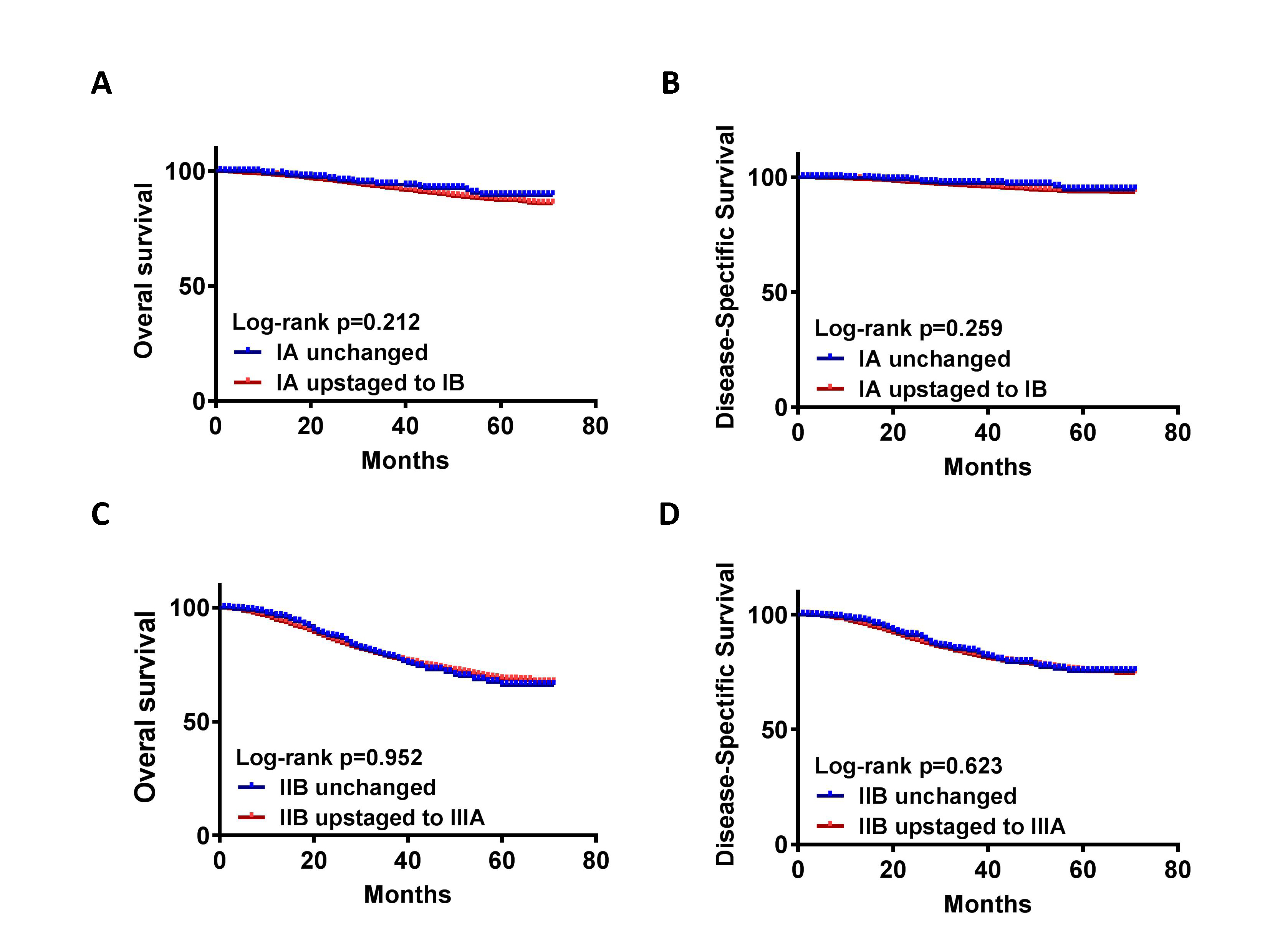

Supplement: Supplementary file 2 — Additional file 2: Figure S2. Kaplan-Meier curves of OS (A) and DSS (B) for the SEER cohort between patients with IA unchanged and those with anatomical stage IA upstaged to prognostic stage IB; Kaplan-Meier curves of OS (C) and DSS (D) for the SEER cohort between patients with IIB unchanged and those with anatomical stage IIB upstaged to prognostic stage IIIA. [file 12885_2019_6494_MOESM2_ESM.jpg]
