# Supplementary material for: AJCC 8th edition prognostic staging provides no better discriminatory ability in prognosis than anatomical staging in triple negative breast cancer
Source: BMC Cancer. 2020 Jan 6;20:18. doi: 10.1186/s12885-019-6494-3 (PMC6945658; doi:10.1186/s12885-019-6494-3)
Supplement: Supplementary file 3 — Additional file 3: Table S1. Clinicopathologic characteristics of TNBCs included in SYSUCC-PWH cohort (N = 611) and SEER cohort (N = 31,941). Table S2. Alteration from Anatomic to Prognostic Stage of TNBCs included in SYSUCC-PWH cohort (N = 611) and SEER cohort (N = 31,941). Table S3. Hazard Ratio for Disease-Specific Survival and Progression-Free Survival by Stage of TNBCs included in the SYSUCC-PWH cohort (N = 611). Table S4. Hazard Ratio for Disease-Specific Survival and Overall Survival by Stage of TNBCs included in the SEER cohort (N = 31,941). [file 12885_2019_6494_MOESM3_ESM.docx]

**Table S1.** Clinicopathologic characteristics of TNBCs included in SYSUCC-PWH cohort (N=611) and SEER cohort (N=31941)

| **Parameters** | **N (%) /SYSUCC-PWH cohort** | **N (%)/SEER cohort** |
| --- | --- | --- |
| **Age** |  |  |
| ≤ 45 years | 210 (34.4%) | 5223 (16.4%) |
| 46-60 years | 289 (47.3%) | 11675 (36.5%) |
| > 60 years | 112 (18.3%) | 15043 (47.1%) |
| **Gender** |  |  |
| Female | 611(100%) | 31915 (99.9%) |
| Male | 0(0.0%) | 26 (0.1%) |
| **Laterality** |  |  |
| Left | 335 (54.8%) | 16435 (51.5%) |
| Right | 267 (43.7%) | 15504 (48.5%) |
| Bilateral  **Location** | 8 (1.3%) | 2 (1.3%) |
| Upper outer quadrant | 256 (41.9%) | 12094 (37.9%) |
| Upper inner quadrant | 82 (13.4%) | 4219 (13.2%) |
| Lower outer quadrant | 51 (8.3%) | 2240 (7.0%) |
| Lower inner quadrant | 24 (3.9%) | 1901 (6.0%) |
| Overlapping quadrant | 132 (21.6%) | 7087 (22.2%) |
| Central | 29 (4.7%) | 1059 (3.3%) |
| Nipple | 11 (1.8%) | 71 (0.2%) |
| Axillary tail | 2 (0.3%) | 237 (0.7%) |
| Unknown | 24 (3.9%) | 2933 (9.2%) |
| **Histological type** |  |  |
| IDC, NOS | 555 (90.8%) | 28034 (87.8%) |
| ILC | 8 (1.3%) | 397 (1.3%) |
| Medullary Carcinoma | 23 (3.8%) | 417 (1.3%) |
| Metaplastic Carcinoma | 11 (1.8%) | 915 (2.9%) |
| Mucinous Carcinoma | 5 (0.8%) | 35 (0.1%) |
| Invasive Micropapillary Carcinoma | 2 (0.3%) | 48 (0.2%) |
| Apocrine Carcinoma | 4 (0.7%) | 351 (1.1%) |
| Mixed Carcinoma | 3 (0.5%) | 1325 (4.2%) |
| Other | 0 (0.0%) | 419 (1.3%) |
| **Anatomical stages (AJCC 7th)** |  |  |
| IA | 100 (16.4%) | 12293 (38.5%) |
| IB | 0 (0.0%) | 407 (1.3%) |
| IIA | 240 (39.3%) | 9713 (30.4%) |
| IIB | 131 (21.4%) | 4579 (14.3%) |
| IIIA | 78 (12.8%) | 2515 (7.9%) |
| IIIB | 29 (4.7%) | 1164 (3.6%) |
| IIIC | 33 (5.4%) | 1270 (4.0%) |
| **Prognostic stages (AJCC 8th)** |  |  |
| IA | 3 (0.5%) | 407 (1.3%) |
| IB | 97 (15.9%) | 11886 (37.2%) |
| IIA | 240 (39.3%) | 10120 (31.7%) |
| IIB | 51 (8.3%) | 606 (1.9%) |
| IIIA | 83 (13.6%) | 3986 (12.5%) |
| IIIB | 26 (4.3%) | 365 (1.1%) |
| IIIC | 111 (18.2%) | 4571 (14.3%) |
| **Histologic grade** |  |  |
| 1 | 7 (1.1%) | 580 (1.8%) |
| 2 | 303 (49.6%) | 5661 (17.7%) |
| 3 | 301 (49.3%) | 25700 (80.5%) |
| **Surgery type** |  |  |
| Breast-conserving surgery | 81 (13.3%) | NA* |
| Mastectomy | 346 (56.6%) | NA |
| Unknown | 184 (30.1%) | NA |
| **Chemotherapy** |  |  |
| Yes | 439 (71.8%) | 25764 (80.7%) |
| No/Unknown | 172 (28.1%) | 6177 (19.3%) |
| **Radiotherapy** |  |  |
| Yes | 103 (16.9%) | 16704 (52.3%) |
| No/Unknown | 508 (83.1%) | 15237 (47.7%) |
| **Progression** |  |  |
| Yes | 132 (21.6%) | NA* |
| No | 478 (78.2%) | NA |
| **Dead of tumor** |  |  |
| Yes | 42 (6.9%) | 3238 (10.1%) |
| No | 567 (92.8%) | 28703 (89.9%) |

|  |
| --- |
|  |
|  |
|  |
|  |
|  |

TNBC, triple negative breast cancer; IDC, invasive ductal carcinoma; NOS, not otherwise specified; ILC, invasive lobular carcinoma; AJCC, American Joint Committee on Cancer. * Data cannot access.

| **Table S2.** Alteration from Anatomic to Prognostic Stage of TNBCs included in SYSUCC-PWH cohort (N=611) and SEER cohort (N=31941) | | |
| --- | --- | --- |
| **Stage alteration** | **N (%) /SYSUCC-PWH cohort** | **N (%) /SEER cohort** |
|  |  |  |
| **AS IA → PS IB** | 97 (15.9%) | 11886 (37.2%) |
| **AS IB → PS IIA** | 0 (0.0%) | 407 (1.3%) |
| **AS IIB → PS IIIA** | 80 (13.1%) | 3973 (12.4%) |
| **AS IIIA → PS IIIB** | 25 (4.1%) | 349 (1.1%) |
| **AS IIIA → PS IIIC** | 50 (8.2%) | 2153 (6.7%) |
| **AS IIIB → PS IIIC** | 28 (4.6%) | 1156 (3.6%) |
| **AS IIIC → PS IIIB** | 0 (0.0%) | 8 (<0.1%) |
|  |  |  |

TNBC, triple negative breast cancer; AS, anatomic stage; PS, prognostic stage

| **Table S3.** Hazard Ratio for Disease-Specific Survival and Progression-Free Survival by Stage of TNBCs included in the SYSUCC-PWH cohort (N=611) | | | | | | | | | | | | | | | | |  |  |
| --- | --- | --- | --- | --- | --- | --- | --- | --- | --- | --- | --- | --- | --- | --- | --- | --- | --- | --- |
| **Stage** | **Anatomic Stage(DSS)** | | **Prognostic Stage(DSS)** | | | | **Anatomic Stage(PFS)** | | | | | **Prognostic Stage(PFS)** | | | | | |  |
|  | **HR (95% CI)** | **p-value** | | **HR (95% CI)** | **p-value** | | | | **HR (95% CI)** | **p-value** | | | | **HR (95% CI)** | **p-value** | | | |
| I* | 1 (Reference) |  | | 1 (Reference) | |  | | 1 (Reference) | | |  | | 1 (Reference) | | |  | | |
| IIA | 0.74 (0.33-1.65) | 0.464 | | 0.74 (0.33-1.65) | | 0.463 | | 1.00 (0.52-1.89) | | | 0.987 | | 1.00 (0.52-1.89) | | | 0.989 | | |
| IIB | 1.35 (0.60-3.06) | 0.475 | | 1.30 (0.46-3.66) | | 0.620 | | 2.13 (1.12-4.02) | | | 0.020 | | 2.49 (1.20-5.19) | | | 0.015 | | |
| IIIA | 2.34 (1.02-5.35) | 0.044 | | 1.30 (0.53-3.21) | | 0.565 | | 3.73 (1.96-7.10) | | | <0.001 | | 2.00 (1.00-3.99) | | | 0.050 | | |
| IIIB | 5.90 (2.19-15.88) | <0.001 | | 2.32 (0.78-6.92) | | 0.132 | | 4.88 (2.08-11.45) | | | <0.001 | | 2.88 (1.23-6.74) | | | 0.015 | | |
| IIIC | 4.58(1.81-11.56) | 0.001 | | 3.72 (1.74-7.94) | | 0.001 | | 8.15 (4.01-16.55) | | | <0.001 | | 5.29 (2.87-9.76) | | | <0.001 | | |

TNBC, triple negative breast cancer; DSS, disease-specific survival; PFS, progression-free survival; HR, hazard ratio; CI, confidence interval. *No case was classified as anatomic stage IB in this cohort.

| **Table S4.** Hazard Ratio for Disease-Specific Survival and Overall Survival by Stage of TNBCs included in the SEER cohort (N=31941) | | | | | | | | | | | | | | | | | | |
| --- | --- | --- | --- | --- | --- | --- | --- | --- | --- | --- | --- | --- | --- | --- | --- | --- | --- | --- |
| **Stage** | **Anatomic Stage(DSS)** | | | **Prognostic Stage(DSS)** | | | | **Anatomic Stage(OS)** | | | | | **Prognostic Stage(OS)** | | | | |  |
|  | **HR (95% CI)** | **p-value** | **HR (95% CI)** | | **p-value** | | **HR (95% CI)** | | **p-value** | | | **HR (95% CI)** | | | **p-value** | | |  |
| IA | 1 (Reference) |  | 1 (Reference) | | |  | 1 (Reference) | | | |  | | | 1 (Reference) | | |  |  |
| IB | 2.42 (1.69-13.46) | <0.001 | 1.43 (0.76-2.68) | | | 0.264 | 1.55 (1.14-2.11) | | | 0.005 | | | | 1.30 (0.86-1.97) | | 0.215 | |  |
| IIA | 2.45 (2.17-2.77) | <0.001 | 3.46 (1.86-6.46) | | | <0.001 | 1.85 (1.69-2.02) | | | <0.001 | | | | 2.36 (1.56-3.57) | | <0.001 | |  |
| IIB | 4.57 (4.03-5.18) | <0.001 | 6.27 (3.25-12.12) | | | <0.001 | 2.95 (2.68-3.24) | | | <0.001 | | | | 3.87 (2.47-6.07) | | <0.001 | |  |
| IIIA | 8.07 (7.10-9.17) | <0.001 | 6.50 (3.48-12.14) | | | <0.001 | 4.79 (4.33-5.30) | | | <0.001 | | | | 3.79 (2.50-5.74) | | <0.001 | |  |
| IIIB | 15.54 (13.53-17.85) | <0.001 | 9.64 (4.97-18.71) | | | <0.001 | 9.32 (8.35-10.41) | | | <0.001 | | | | 5.46 (3.46-8.63) | | <0.001 | |  |
| IIIC | 16.38 (14.35-18.70) | <0.001 | 17.00 (9.13-31.68) | | | <0.001 | 9.20 (8.27-10.23) | | | <0.001 | | | | 9.03 (5.98-13.62) | | <0.001 | |  |

TNBC, triple negative breast cancer; DSS, disease-specific survival; OS, overall survival; HR, hazard ratio; CI, confidence interval.
